# Supplementary material for: Risk Factors for Therapeutic Intervention of Remdesivir in Mild to Moderate COVID-19—A Single-Center Retrospective Study of the COVID-19 Fourth Pandemic Period in Wakayama, Japan
Source: Medicina (Kaunas). 2022 Jan 13;58(1):118. doi: 10.3390/medicina58010118 (PMC8779147; doi:10.3390/medicina58010118)
Supplement: Supplementary file 1 [file medicina-58-00118-s001.zip › medicina-1470945-supplementary.pdf]

Patient Questionnaire (Symptoms related to COVID-19 infection)

Date:____________

Name:______________________ Age:________

Address:__________________________ Date of Admission:_____________

Please answer the following questions:

1) Do you have runny nose?	◻ Yes	◻ No
2) Do you have throat pain?	◻ Yes	◻ No
3) Do you have cough?	◻ Yes	◻ No
4) Do you have sputum?	◻ Yes	◻ No
5) Do you have general fatigue?	◻ Yes	◻ No
6) Do you have headache?	◻ Yes	◻ No
7) Do you have arthralgia?	◻ Yes	◻ No
8) Do you have gastrointestinal symptoms?	◻ Yes	◻ No
9) Do you have loss of smell?	◻ Yes	◻ No
10) Do you have loss of taste?	◻ Yes	◻ No
11) Do you have shortness of breath?	◻ Yes	◻ No

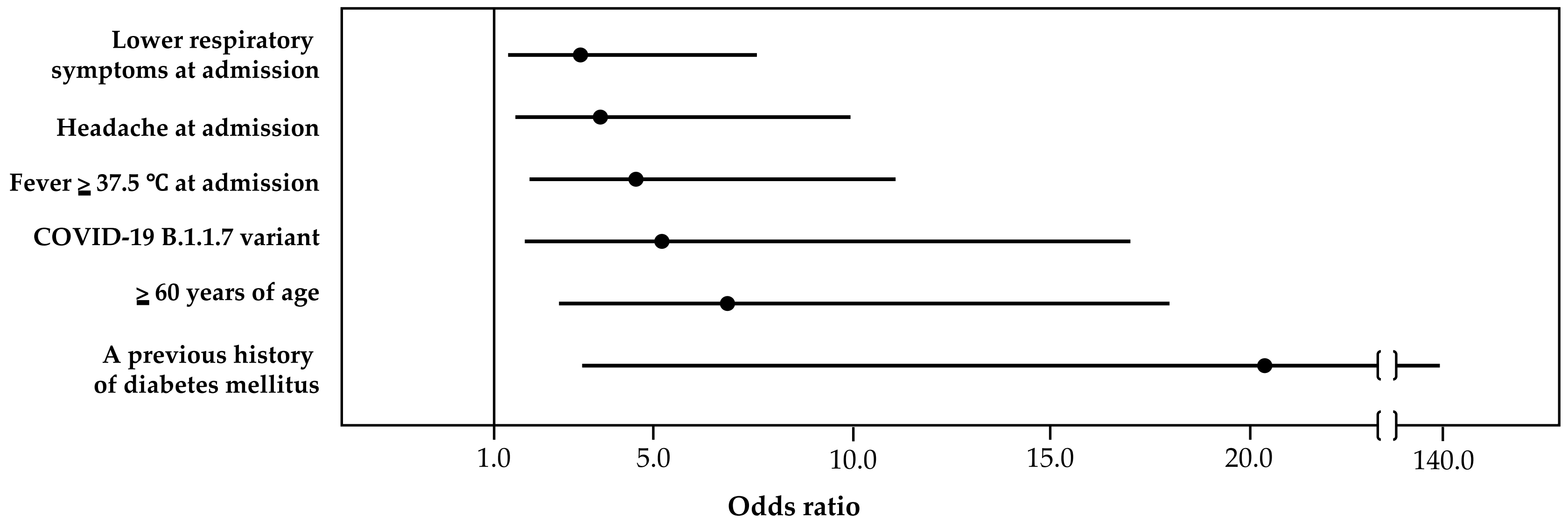

Supplemental Figure S1. Adjusted OR of risk factors for therapeutic intervention of remdesivir among mild to moderate COVID-19 hospitalized patients. Circles represent adjusted OR, error bars represent 95% CI.
